# A Few Thoughts on the Study and Practice of Homœopathy

**Published:** 1882-05

**Authors:** David Wilson

**Affiliations:** London, England


### A FEW THOUGHTS ON THE STUDY AND PRACTICE OF HOMŒOPATHY.

(*From the Transactions of the World's Homœopathic Convention, held in Philadelphia*, 1876.)

DAVID WILSON, M. D., LONDON, ENGLAND.

Deeply sensible of the honor conferred upon me by the courteous invitation of the Chairman of the Committee of Arrangements, to contribute a paper for the "World's Homœopathic Convention," I must frankly confess that it roused in my mind a question which has forced itself upon me daily since, viz.: What can be said or done to advance the cause of Homœopathy better than the luminous instruction contained in the writings of Hahnemann and the untiring exertions of his early disciples, some of whom are still working amongst us? This reflection naturally suggests the pertinent inquiry: Have Hahnemann's works been studied as they require to be? and what evidence have we, affirmatory or otherwise, that enables us to answer the question? It would be false to the principles which I profess were I not, *in limine*, openly to state my opinions fully and candidly upon a subject of such vital importance.

To sustain my proposition, it will be necessary to quote from our great master's writings, for which no apology will be necessary, seeing that when our object is solely the elucidation of truth it is far better to go to the fountain-head at once, than to assume an originality not our own.

Hahnemann tells us, as we all ought to remember, that his doctrine of Homœopathy rests exclusively upon the result of experience. He tells us in his *Organon* how we are to apply his doctrine in practice so as to gain pure experience. He tells us in the same invaluable *Organon* how we are to proceed in acquiring a knowledge of medicinal agents, so that they may be used as remedies in healing the sick; in other words, he instructs us how we are to proceed in proving remedies upon ourselves and others, when in ordinary health. He points out clearly in the same work, how we are to examine the sick. He enters into many other important matters in that essential work which must be studied attentively from board to board, again and again, until every precept has been thoroughly understood and impressed upon the mind for our guidance when entering upon the responsible and solemn duty of ministering to the sick. I repeat, there can be no flinching from this absolute moral duty; and if we cannot conscientiously comply with Hahnemann's injunctions, then let us not falsely call ourselves *his* disciples.

The learned chairman, Dr. Carroll Dunham, has most forcibly remarked, in an article published in the *Philadelphia Journal of Homœopathy*, vol. iv, 1855, No. 8, which I should recommend every one to read even a second and third time if they have not already done so: "When a scientific man undertakes to imitate the experiment of another for the purpose of testing the results of the latter, he is inexcusable if he deviates in the least particular from the course pursued by his predecessor. He has no right to assume that in this or that point lies the essential principle, and that the other matters are superfluous. What would be thought of a chemist who, wishing to repeat the experiments of another, and obtain a certain precipitate, should take it for granted that it matters not how much ammonia, for example, he may add to a given solution, whereas, a certain quantity will give the precipitate, but an excess will re-dissolve it and spoil the experiment." Nothing could be said more conclusively in support of our argument than this.

Now, let us hear what Hahnemann says: "Imitate my mode of practice accurately and carefully, as pointed out in the *Organon* and *Chronic Diseases*, and you will find it confirmed at every step." He says further: "Take one case of disease after another, note down all of its perceptible symptoms in the special manner pointed out in the *Organon*, and with such accuracy as will defy the founder of Homœopathy to take any exception to it; then, guided by the *characteristic* and *striking* symptoms, select the appropriate remedy and administer it in the smallest dose, according to the strict rules and observances pointed out in the *Organon*, and if it does not afford speedy, gentle, lasting help, publish the failure to the world, and the doctrine of Homœopathy shall stand abashed." He urges upon us, who are his disciples, to go on proving remedies, so that the range of our curative means shall be equal to every emergency, because it is needful, as a matter of course, before we can prescribe for every particular ailment, that we shall be acquainted with a corresponding remedy, the symptoms of which are harmonic or homœopathic to those of the patient.

Then, with regard to the infinitesimal character of the dose and its repetition, his injunctions are equally imperative, while he wisely makes a provision for every emergency, so that the dose may be repeated in the highest dilutions every five minutes if needful. His rules, as laid down in the *Organon*, for the discovery of commencing improvement and how *then* to proceed, are pointed out with unerring certainty, and must be very carefully studied. As we ponder over Hahnemann's writings, not forgetting his instructive introductions to his remedies, we soon discover *why* he gradually reduced his doses, chiefly because they acted too energetically, and inflicted unnecessary suffering and exhaustion.

While imbibing all these facts and practical details, let the student keep constantly in remembrance that he has to separate Hahnemann's facts from his theories, a caution which the founder himself thought it needful to give, but which his ungenerous and carping critics have pounced upon as capital for their unbecoming ridicule, being unable to overthrow his facts, which stand as firm as the ocean rock, though lashed on all sides by the waves of ignorance and superstition! He says in vol. iv of *Chronic Diseases:* "In bringing forward my doctrine of the homœopathic system of healing, it was very natural for me not to venture an explanation *how* it happens that cures are effected in the sick through the action of certain substances that have the power of producing very similar morbid phenomena in healthy people. It was with a feeling of doubt that I gave my conjecture, without attempting anything like an explanation or asserting anything positive, for it is only incumbent upon us to heal according to certain recognized laws of nature (as expounded in the *Organon* and *Chronic Diseases*), which are being continually confirmed by our experience, and not to make a boast of abstract explanations and leave the patient uncured all the while, a mode of proceeding hitherto adopted by physicians of the old school." It will not, I trust, be considered presumptuous on my part if I give my most cordial assent to the close of Hahnemann's sentence, seeing that I was an active member amongst the old fraternity for many years, sufficient to enable me to superintend not less than two thousand births in general practice; and the last four years of my allopathic practice afforded me the opportunity to test Homœopathy, to which I have devoted the last thirty years of my life in hard study of its materia medica and active practice.

Now it may be asked what evidence have we that the majority, if not all, calling themselves homœopaths have failed to comply with Hahnemann's injuctions? The answer is to be found in the character of homœopathic literature, and in the majority of the editorial staff, who do not fail to chide the pure Hahnemannians as being, on this side of the Atlantic at least, a very infinitesimal sect of Hahnemannians. What proof could we have more conclusive than this? They seemingly forget, however, that "minorities are often in the right." Then with regard to modern, so-called provings, have the injunctions so specially laid down in the *Organon* been complied with? I answer most emphatically no, they have not; and if we examine carefully the records of the Vienna and other provers, we shall find Hahnemann's predictions verified when he says, § 128, and following: "The most recent observations have shown that medicinal substances, when taken in their crude state for the purpose of testing their peculiar effects, do not exhibit nearly the full amount of their hidden powers as they do when potentized by proper trituration and succussion. We now find it best to prove medicines by giving 4 to 6 globules of the 30th dilution every morning on an empty stomach. * * * If the effects are but slight, we may add a few more globules every morning, until the symptoms become more distinct and stronger. If the first dose administered has been sufficiently strong to arouse symptoms, then the experimenter learns the order of their succession and can accurately note the period when each occurs, which is very useful in leading to a knowledge of the genius of the medicine, and the order of the primary and alternating actions is acquired in the most unambiguous manner. * * *

On the other hand, if increasing doses of a strong preparation are repeated (as was the case in most of the Vienna provers), we may learn, no doubt, the various morbid states such strong doses are capable of producing, but we do not ascertain their order of succession, and the *subsequent dose often removes, curatively*, some one or other of the symptoms aroused by the previous dose, or develops in its place an opposite state." What has been here quoted in a condensed, but nevertheless strictly accurate manner, plainly shows the chaos into which some of our would-be heroic provers have plunged the materia medica since Hahnemann's time. Without mentioning the names of *such* provers, which might seem discourteous, and would render very little service, every industrious and careful student can discover for himself these blemishes to which I have alluded. Also the fact mentioned by Hahnemann has been verified, for when the same prover substituted for his massive and poisonous doses dilutions of the same remedy, then he obtained finer shades of minute symptoms, such as are to be observed in disease. It becomes a question, however, whether the symptoms furnished by provers through taking attenuated doses almost immediately after they had endeavored to poison themselves without success by massive and crude doses, are to be entirely relied upon. For example, one would scarcely consider a man who had taken in sixty-eight days the enormous quantity of *five thousand* drops of a poisonous tincture, in a perfectly healthy condition, so as to resume, after a few days' cessation, the proving of the same remedy in diluted doses. We are cautioned by Hahnemann to examine into the perfect purity of substances before we undertake their proving as remedies, and, while we are under medical influences, to abstain from other experiments and repetitions that may annul the integrity of our observations. As to the time needful for the organism to regenerate itself, I must leave others more capable to determine, without entering into the biological and mathematical speculation myself, of that process being accomplished in 35 days, the time required, according to Grauvogl, for a proving to have its effects observed, under the most varied circumstances possible, upon persons of various ages, constitutions, etc. He adds that it is "only after the fulfillment of all these demands that we can say we have undertaken a drug proving and gained available results." We have it in evidence too clearly, however, that whatever may be the length of time for the organism to reconstruct itself, the interchanges do not, unfortunately, expel for a very long time many substances that have entered it by design or otherwise. Grauvogl himself, indeed, tells us that "there are substances which, when introduced into the organism in immoderate quantities, are suddenly rendered innocuous by their entering into insoluble compounds, which remain deposited in some part or other, and thus become inaccessible to the motions of the organism, and continue to be so, till some other substance, introduced accidentally or intentionally into the organism, enters into combination with the very same organic parts in which those substances are deposited, and thus, made accessible again to the organic motion, they may forthwith be expelled from the system." Such substances may be of a character to be detected by the acute analyst in company with the various excretions. Quicksilver, for example, may be detected, chemico-physically, in the urine after many years, during which period it may now and again have given rise to a series of suffering phenomena. Such cases falling into the hands of the over-enthusiastic tyros of our system, may easily lead them to undertake cures no longer possible, and therefore calculated to inflict cruel disappointment upon those most deeply concerned. Such cases require antidotal and eliminative treatment to begin with, as ably pointed out by Dr. Lippe in an admirable article entitled "Homœopathy Misapplied," in the *North American Journal of Homœopathy*, 1872. If we wish to *test* Hahnemann let us abide strictly by his rules in every essential particular, and afterwards we may indulge in our own theories.

[CONCLUDED IN NEXT ISSUE.]

